# Supplementary material for: Klebsiella oxytoca facilitates microbiome recovery via antibiotic degradation and restores colonization resistance in a diet-dependent manner
Source: Nat Commun. 2025 Jan 9;16:551. doi: 10.1038/s41467-024-55800-y (PMC11717976; doi:10.1038/s41467-024-55800-y)
Supplement: Supplementary file 1 — Supplementary Information [file 41467_2024_55800_MOESM1_ESM.pdf]

Supplementary Information

Klebsiella oxytoca facilitates microbiome recovery via antibiotic degradation and restores colonization resistance in a diet-dependent manner

Éva d.H. Almási, Lea Eisenhard*, Lisa Osbelt*, Till Robin Lesker, Anna C. Vetter, Nele Knischewski, Agata Anna Bielecka, Achim Gronow, Uthayakumar Muthukumarasamy, Marie Wende, Caroline Tawk, Meina Neumann-Schaal, Mark Brönstrup, Till Strowig

*: These authors contributed equally

Lead contact: Till Strowig, Department of Microbial Immune Regulation, Helmholtz Centre for Infection Research (HZI), 38124 Braunschweig, Germany, till.strowig@helmholtz-hzi.de

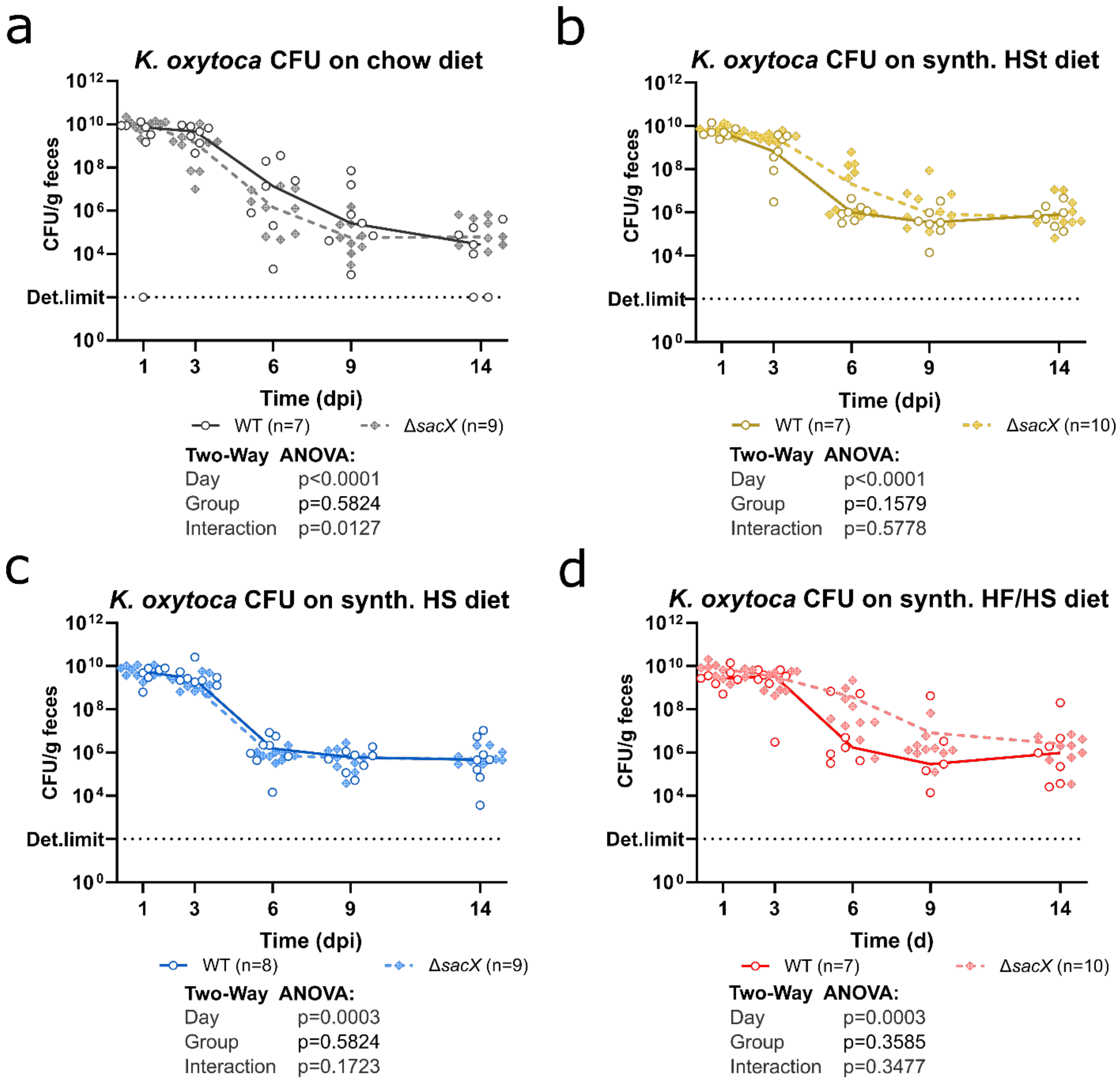

a
K. oxytoca CFU on chow diet
CFU/g feces
Det.limit
Time (dpi)
WT (n=7)
ΔsacX (n=9)
Two-Way ANOVA:
Day p<0.0001
Group p=0.5824
Interaction p=0.0127
b
K. oxytoca CFU on synth. HSt diet
CFU/g feces
Det.limit
Time (dpi)
WT (n=7)
ΔsacX (n=10)
Two-Way ANOVA:
Day p<0.0001
Group p=0.1579
Interaction p=0.5778
c
K. oxytoca CFU on synth. HS diet
CFU/g feces
Det.limit
Time (dpi)
WT (n=8)
ΔsacX (n=9)
Two-Way ANOVA:
Day p=0.0003
Group p=0.5824
Interaction p=0.1723
d
K. oxytoca CFU on synth. HF/HS diet
CFU/g feces
Det.limit
Time (d)
WT (n=7)
ΔsacX (n=10)
Two-Way ANOVA:
Day p=0.0003
Group p=0.3585
Interaction p=0.3477

Supplementary Figure 1: Genetic disruption of sucrose utilization does not affect *K. oxytoca* colonization levels *in vivo* (a-d) CFUs of *K. oxytoca* WT or Δ*sacX* in *K. oxytoca* pre-colonized mice fed **(a)** standard chow, **(b)** semi-synthetic, **(c)** semi-synthetic high-sucrose or **(d)** semi-synthetic high-fat/high-sucrose diets from 2-3 independent experiments with n = 3-5 mice/group. Global p values represent a two-way repeated-measured ANOVA with Geisser-Greenhouse correction with $*p < 0.05$, $**p < 0.01$, $***p < 0.005$, $****p < 0.0001$. Source data are provided as a Source Data file.

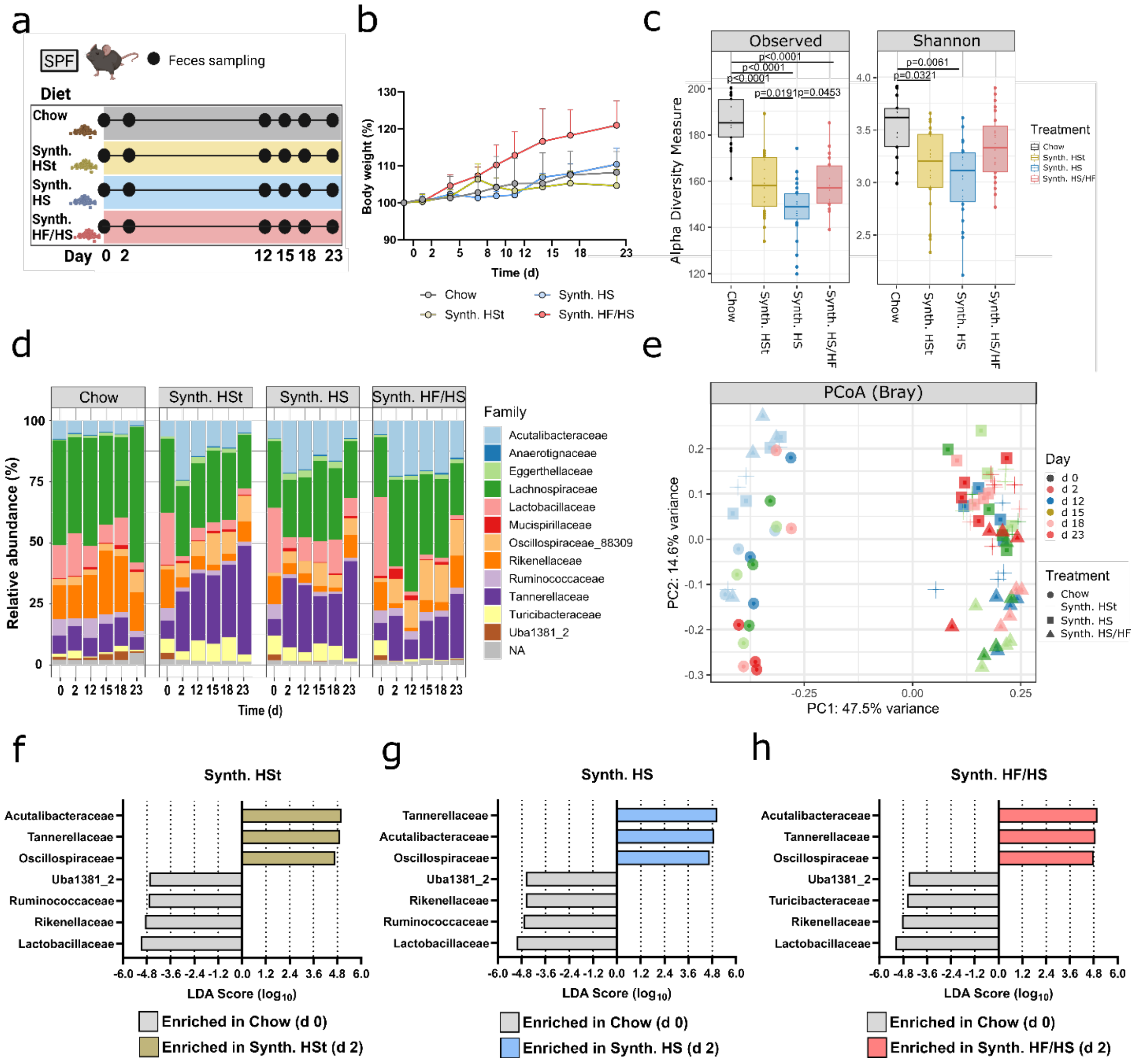
a
b
c
d
e
f
g
h
SPF
Feces sampling
Diet
Chow
Synth. HSt
Synth. HS
Synth. HF/HS
Day 0 2 12 15 18 23
Body weight (%)
Time (d)
Chow
Synth. HSt
Synth. HS
Synth. HF/HS
Observed
Shannon
Alpha Diversity Measure
p<0.0001
p<0.0001
p<0.0001
p=0.0191
p=0.0453
p=0.0061
p=0.0321
Treatment
Chow
Synth. HSt
Synth. HS
Synth. HS/HF
Relative abundance (%)
Family
Acutalibacteraceae
Anaerotignaceae
Eggerthellaceae
Lachnospiraceae
Lactobacillaceae
Mucispirillaceae
Oscillospiraceae_88309
Rikenellaceae
Ruminococcaceae
Tannerellaceae
Turicibacteraceae
Uba1381_2
NA
PCoA (Bray)
PC1: 47.5% variance
PC2: 14.6% variance
Day
d 0
d 2
d 12
d 15
d 18
d 23
Treatment
Chow
Synth. HSt
Synth. HS
Synth. HS/HF
Synth. HSt
Acutalibacteraceae
Tannerellaceae
Oscillospiraceae
Uba1381_2
Ruminococcaceae
Rikenellaceae
Lactobacillaceae
LDA Score (log10)
Enriched in Chow (d 0)
Enriched in Synth. HSt (d 2)
Synth. HS
Tannerellaceae
Acutalibacteraceae
Oscillospiraceae
Uba1381_2
Rikenellaceae
Ruminococcaceae
Lactobacillaceae
LDA Score (log10)
Enriched in Chow (d 0)
Enriched in Synth. HS (d 2)
Synth. HF/HS
Acutalibacteraceae
Tannerellaceae
Oscillospiraceae
Uba1381_2
Turicibacteraceae
Rikenellaceae
Lactobacillaceae
LDA Score (log10)
Enriched in Chow (d 0)
Enriched in Synth. HF/HS (d 2)

Supplementary Figure 2: Low-fiber semi-synthetic diets decrease α-diversity of the gut microbiome and change its composition. (a) Schematic showing experimental setup. All mice were switched to one of four diets on day 0. Fecal samples were collected on the indicated days. **(b)** Mean body weights of mice fed different diets, data points show group means with standard deviation. **(c)** Fecal microbiota α-diversity depicted by observed ASVs and Shannon-scores. P-values represent Tukey's multiple comparison test with *$p < 0.05$, **$p < 0.01$, ***$p < 0.005$, ****$p < 0.0001$. **(d)** Relative abundances of the 12 most abundant families shown as group average for untreated control mice from one experiment with n = 3-4 mice/group. **(e)** β-diversity depicted by a PCoA plot calculated based on Bray-Curtis distances. **(f-h)** Analysis of differentially abundant bacterial families in mice fed **(f)** synthetic, **(g)** synthetic high-sucrose and **(h)** synthetic high-fat/high-sucrose diet by LEfSe. Panel **(a)** Created with BioRender.com. Source data are provided as a Source Data file.

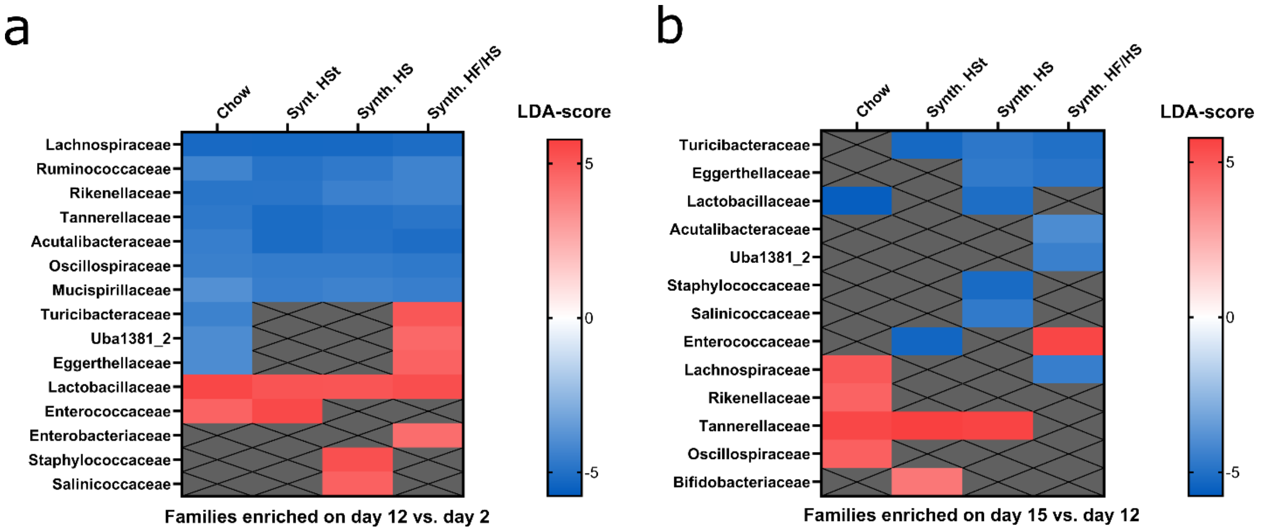
a
Chow
Synt. HSt
Synth. HS
Synth. HF/HS
LDA-score
Lachnospiraceae
Ruminococcaceae
Rikenellaceae
Tannerellaceae
Acutalibacteraceae
Oscillospiraceae
Mucispirillaceae
Turicibacteraceae
Uba1381_2
Eggerthellaceae
Lactobacillaceae
Enterococcaceae
Enterobacteriaceae
Staphylococcaceae
Salinicoccaceae
5
0
-5
Families enriched on day 12 vs. day 2
b
Chow
Synth. HSt
Synth. HS
Synth. HF/HS
LDA-score
Turicibacteraceae
Eggerthellaceae
Lactobacillaceae
Acutalibacteraceae
Uba1381_2
Staphylococcaceae
Salinicoccaceae
Enterococcaceae
Lachnospiraceae
Rikenellaceae
Tannerellaceae
Oscillospiraceae
Bifidobacteriaceae
5
0
-5
Families enriched on day 15 vs. day 12

Supplementary Figure 3: Ampicillin treatment leads to diet-specific compositional changes on the gut microbiome. Analysis of differentially abundant bacterial families in mice (from one experiment with n = 3-4 mice/group) **(a)** at the first recovery time point following the cessation of ampicillin treatment versus at the start of the treatment and **(b)** at the second recovery time point versus the first recovery time point by LEfSe. 15 dpd is equivalent to 6 dpi, when pathogen clearance initiates. Families marked with a black cross indicate no significant enrichment in that given time point. Source data are provided as a Source Data file.

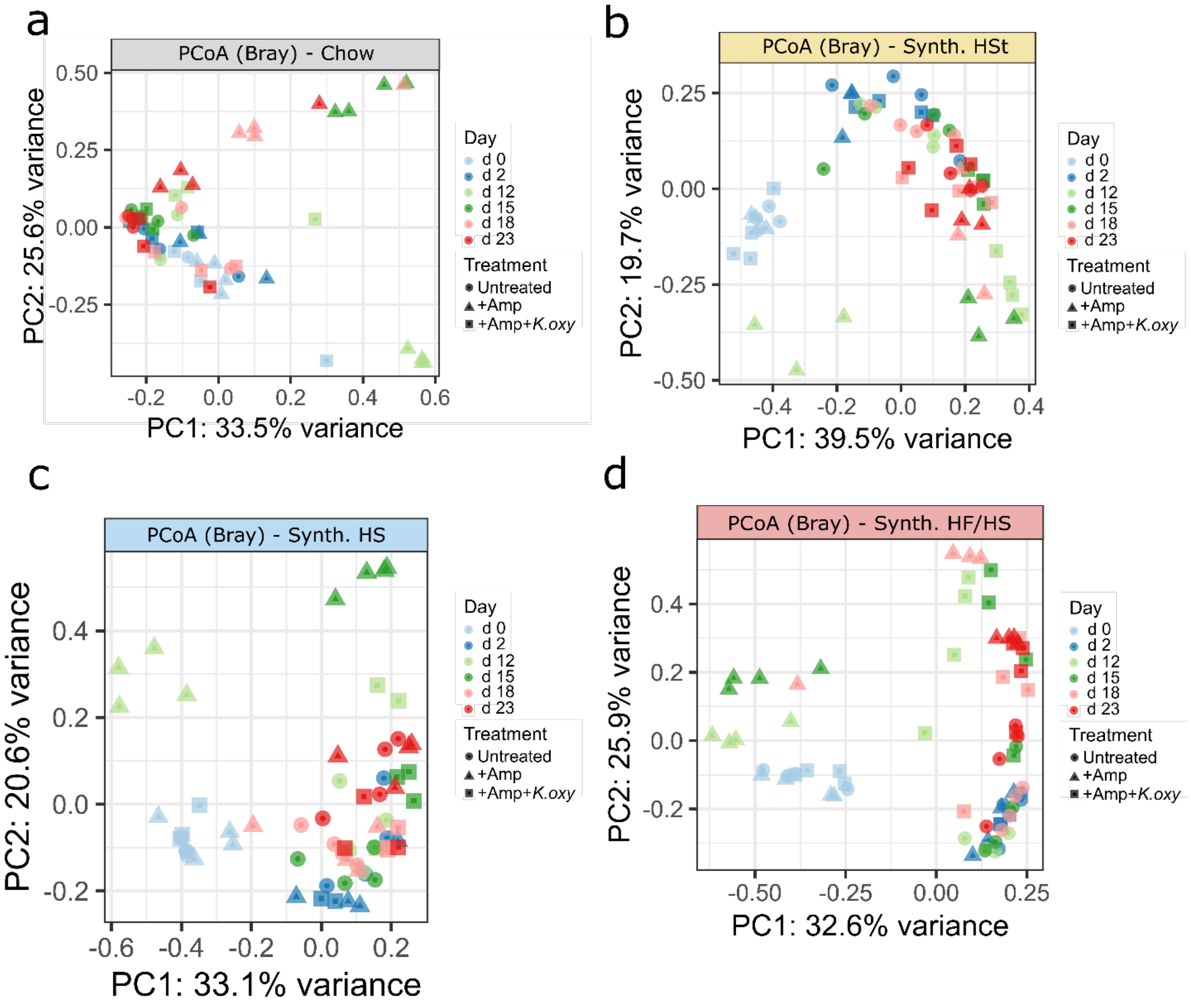
a
PCoA (Bray) - Chow
PC2: 25.6% variance
PC1: 33.5% variance
b
PCoA (Bray) - Synth. HSt
PC2: 19.7% variance
PC1: 39.5% variance
c
PCoA (Bray) - Synth. HS
PC2: 20.6% variance
PC1: 33.1% variance
d
PCoA (Bray) - Synth. HF/HS
PC2: 25.9% variance
PC1: 32.6% variance
Day
d 0
d 2
d 12
d 15
d 18
d 23
Treatment
Untreated
+Amp
+Amp+*K.oxy*

Supplementary Figure 4: *K. oxytoca* colonization facilitates post-ampicillin microbiome recovery: β-diversity based on 16S rRNA gene amplicon sequencing data depicted by a PCoA plot calculated based on Bray-Curtis distances comparing different treatments of mice fed **(a)** standard chow, **(b)** HSt, **(c)** HS and **(d)** HF/HS diets from one experiment with n = 3-4 mice/group. Each symbol represents a fecal sample collected from a single mouse, color indicates time of sampling, shape indicates treatment group. Source data are provided as a Source Data file.

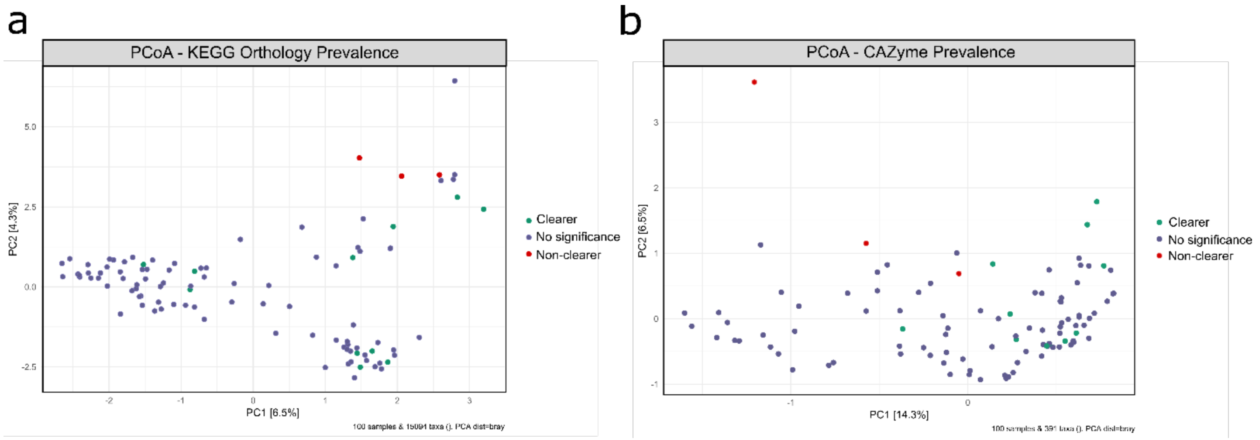
a
PCoA - KEGG Orthology Prevalence
PC2 [4.3%]
5.0
2.5
0.0
-2.5
-2
-1
0
1
2
3
PC1 [6.5%]
Clearer
No significance
Non-clearer
100 samples & 15094 taxa (). PCA dist=bray
b
PCoA - CAZyme Prevalence
PC2 [6.5%]
3
2
1
0
-1
-1
0
PC1 [14.3%]
Clearer
No significance
Non-clearer
100 samples & 391 taxa (). PCA dist=bray

Supplementary Figure 5: Species enriched in “clearer” and “non-clearer” samples encode distinct functional features: Ordination of MAGs filtered by abundance of 0.5% in any selected sample by **(a)** KEGG and **(b)** CAZyme functional profiles. Colors indicate if the feature is discriminative in LEfSe test in “Clearer” or “Non-clearer” samples. Source data are provided as a Source Data file.

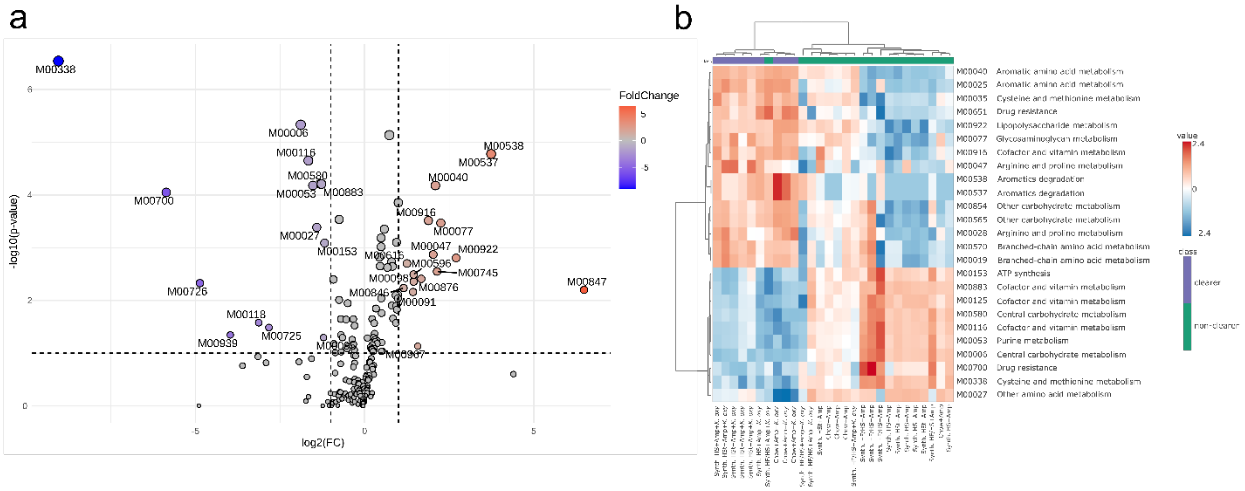
a
b
-log10(p-value)
log2(FC)
FoldChange
M00338
M00006
M00116
M00580
M00053
M00883
M00700
M00027
M00153
M00726
M00118
M00725
M00939
M00538
M00537
M00040
M00916
M00077
M00047
M00922
M00596
M00745
M00876
M00091
M00846
M00847
M00907
M00040 Aromatic amino acid metabolism
M00025 Aromatic amino acid metabolism
M00035 Cysteine and methionine metabolism
M00651 Drug resistance
M00922 Lipopolysaccharide metabolism
M00077 Glycosaminoglycan metabolism
M00916 Cofactor and vitamin metabolism
M00047 Arginine and proline metabolism
M00538 Aromatics degradation
M00537 Aromatics degradation
M00854 Other carbohydrate metabolism
M00565 Other carbohydrate metabolism
M00028 Arginine and proline metabolism
M00570 Branched-chain amino acid metabolism
M00019 Branched-chain amino acid metabolism
M00153 ATP synthesis
M00883 Cofactor and vitamin metabolism
M00125 Cofactor and vitamin metabolism
M00580 Central carbohydrate metabolism
M00116 Cofactor and vitamin metabolism
M00053 Purine metabolism
M00006 Central carbohydrate metabolism
M00700 Drug resistance
M00338 Cysteine and methionine metabolism
M00027 Other amino acid metabolism
value
2.4
0
2.4
class
clearer
non-clearer

Supplementary Figure 6: KEGG functional groups in "clearer" and "non-clearer" samples: (a) The volcano plot shows the significantly differentially-enriched KEGG profiles with a log2 fold-change > 1, **(b)** the heatmap depicts the metagenomic representation of the top 25 significantly differentially enriched KEGG groups based on a two-sample t-test ($p < 0.05$). Colors represent log10 transformed, mean-centered TPM data divided by the standard deviation of each variable. Source data are provided as a Source Data file.

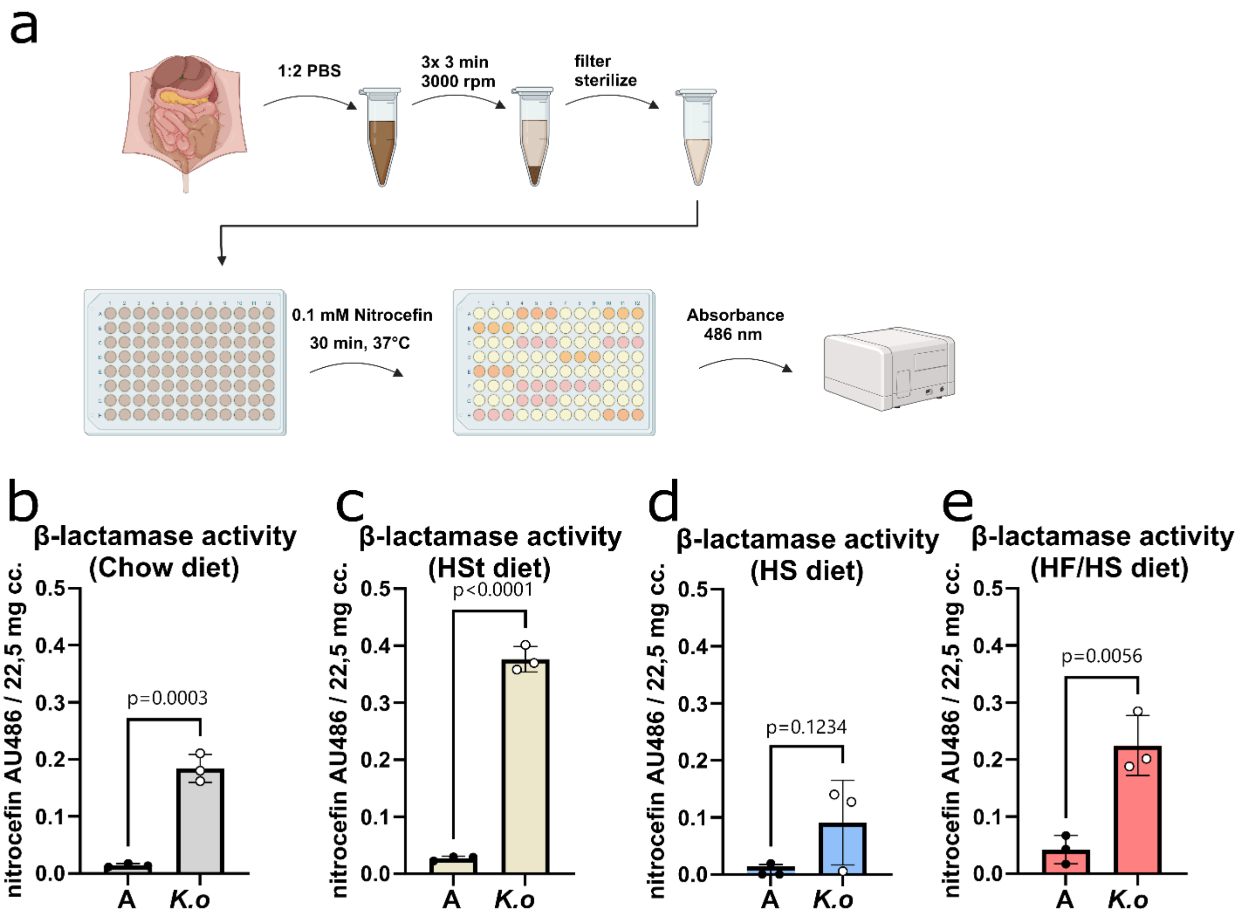
a
1:2 PBS
3x 3 min
3000 rpm
filter
sterilize
0.1 mM Nitrocefin
30 min, 37°C
Absorbance
486 nm
b
β-lactamase activity
(Chow diet)
p=0.0003
c
β-lactamase activity
(HSt diet)
p<0.0001
d
β-lactamase activity
(HS diet)
p=0.1234
e
β-lactamase activity
(HF/HS diet)
p=0.0056
nitrocefin AU486 / 22,5 mg cc.
0.0
0.1
0.2
0.3
0.4
0.5
A
K.o

Supplementary Figure 7: Murine caecum content collected from *K. oxytoca* precolonized mice exerts β-lactamase activity *ex vivo* (a) Schematic showing experimental setup. Mice from one experiment with n = 3 mice/group were sacrificed and their caecum content was isolated and diluted in PBS 1:2, followed by three rounds of centrifugation and sterile filtering the caecum content supernatant **(b)-(e)** *Ex vivo* β-lactamase activity in murine caecum content of mice fed the indicated diets with or without *K. oxytoca* colonization. Colorimetric reaction of 5 µl of β-lactam nitrocefin 30 minutes after spiked into 45 µl filter sterilized caecum contents supernatants diluted with PBS 1:2. Each dot represents the mean of three technical replicates from individual mice. Mean values were normalized to mean values measured at the start of the experiment. The indicated p-values represent unpaired t-test with $^*p < 0.05$, $^{**}p < 0.01$, $^{***}p < 0.005$, $^{****}p < 0.0001$. Panel **(a)** Created with BioRender.com. Source data are provided as a Source Data file.

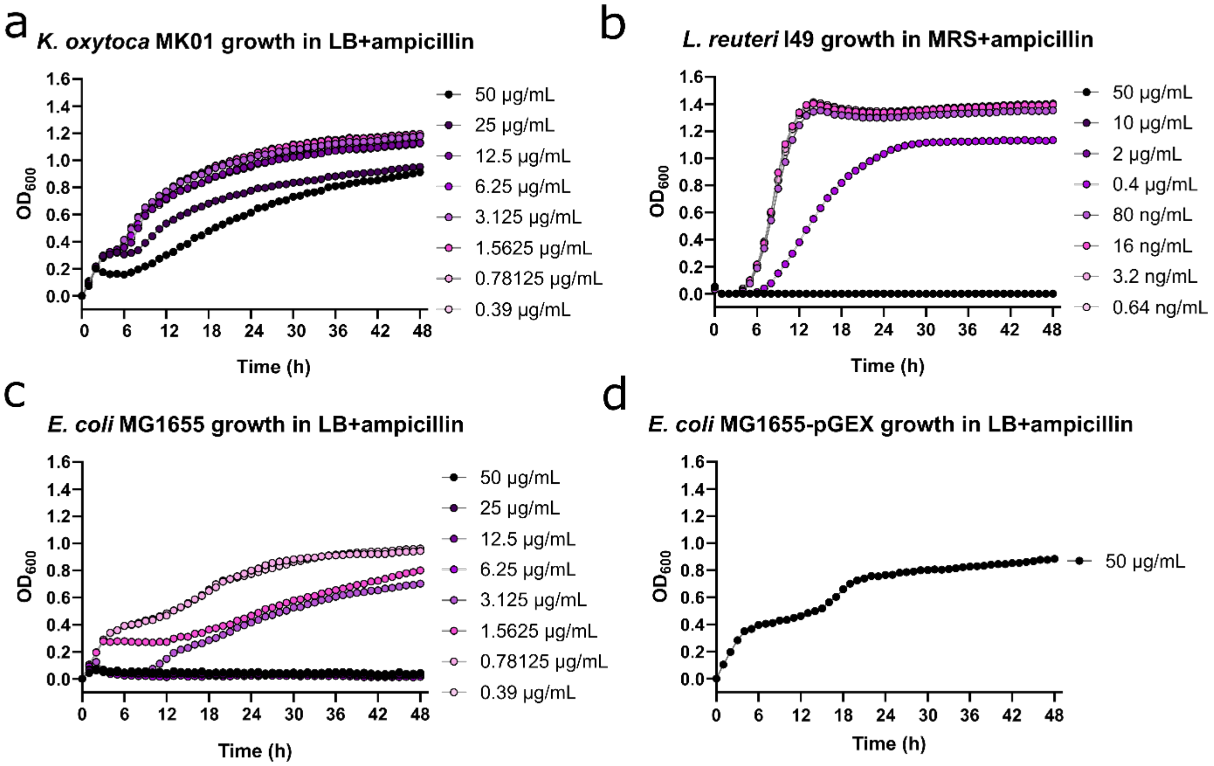
a
K. oxytoca MK01 growth in LB+ampicillin
b
L. reuteri I49 growth in MRS+ampicillin
c
E. coli MG1655 growth in LB+ampicillin
d
E. coli MG1655-pGEX growth in LB+ampicillin
OD600
Time (h)
50 μg/mL
25 μg/mL
12.5 μg/mL
6.25 μg/mL
3.125 μg/mL
1.5625 μg/mL
0.78125 μg/mL
0.39 μg/mL
10 μg/mL
2 μg/mL
0.4 μg/mL
80 ng/mL
16 ng/mL
3.2 ng/mL
0.64 ng/mL

Supplementary Figure 8: *K. oxytoca*, *L. reuteri* I49 and *E. coli* MG1655 show differential sensitivity to ampicillin: Aerobic growth of (a) *L. reuteri* I49, (b) *K. oxytoca* MK01, (c) *E. coli* MG1655 WT and (d) *E. coli* MG1655+pGEX on different concentration of ampicillin supplemented to rich media, MRS for *L. reuteri* I49 and LB for *K. oxytoca* MK01 and *E. coli* MG1655. Dots represent mean OD_{600} values of three technical replicates. Source data are provided as a Source Data file.
