# Supplementary material for: Klebsiella oxytoca facilitates microbiome recovery via antibiotic degradation and restores colonization resistance in a diet-dependent manner
Source: Nat Commun. 2025 Jan 9;16:551. doi: 10.1038/s41467-024-55800-y (PMC11717976; doi:10.1038/s41467-024-55800-y)
Supplement: Supplementary file 2 — Description of Additional Supplementary Files [file 41467_2024_55800_MOESM2_ESM.pdf]

<u>**Description of Additional Supplementary Files**</u>

Supplementary Data 1: Differentially abundant bacterial taxa and carbohydrate active enzymes (CAZymes) associated with *K. pneumoniae* clearance or its absence: (Sheet 1) Sample metadata and assignment into either 'clearer' or 'non-clearer' groups. **(Sheet 2-3)** Output of LEfSe analysis of significantly enriched **(Sheet 2)** bacterial taxa (from de novo generated MAG profiles) and **(Sheet 3)** CAZymes.

Supplementary Data 2: Ampicillin measurements in murine caecal samples with LC-MS/MS: (Sheet 1) MRM transitions of ampicillin, caffeine and trimethoprim. **(Sheet 2)** Raw LC-MS/MS data of ampicillin measured from murine caecal samples.

Supplementary Data 3: Differentially abundant KEGG modules associated with *K. pneumoniae* clearance or its absence: (Sheet 1) Raw data of volcano plot showing KEGG profiles with a log2 fold-change > 1. **(Sheet 2)** Raw data of significantly differentially enriched KEGG modules based on a two-sample t-test.

Supplementary Data 4: List of number and sex of all experimental animals used in the study: Each sheet represents the figure(s) in which data the animal experiment is visualized. If data from the same animal experiment was visualized on separate figures, it is indicated in the name of the data sheet. If data from

multiple independent experiments was pooled and visualized together, it is indicated as separate columns. M = male; F = female mouse.

Supplementary Data 5: Commercially available diets used in the study: Details of the nutritional composition of standard chow, semi-synthetic high-starch, semi-synthetic high-sucrose and semi-synthetic high-fat/high-sucrose diets of Ssniff Spezialdiäten.
