# Supplementary material for: Klebsiella oxytoca facilitates microbiome recovery via antibiotic degradation and restores colonization resistance in a diet-dependent manner
Source: Nat Commun. 2025 Jan 9;16:551. doi: 10.1038/s41467-024-55800-y (PMC11717976; doi:10.1038/s41467-024-55800-y)
Supplement: Supplementary file 8 — Reporting Summary [file 41467_2024_55800_MOESM8_ESM.pdf]

nature portfolio

Corresponding author(s): Till Strowig

Last updated by author(s): Nov 29, 2024

Reporting Summary

Nature Portfolio wishes to improve the reproducibility of the work that we publish. This form provides structure for consistency and transparency in reporting. For further information on Nature Portfolio policies, see our Editorial Policies and the Editorial Policy Checklist.

Statistics

For all statistical analyses, confirm that the following items are present in the figure legend, table legend, main text, or Methods section.

n/a	Confirmed	
☐	☒	The exact sample size (*n*) for each experimental group/condition, given as a discrete number and unit of measurement
☐	☒	A statement on whether measurements were taken from distinct samples or whether the same sample was measured repeatedly
☐	☒	The statistical test(s) used AND whether they are one- or two-sided<br>*Only common tests should be described solely by name; describe more complex techniques in the Methods section.*
☒	☐	A description of all covariates tested
☐	☒	A description of any assumptions or corrections, such as tests of normality and adjustment for multiple comparisons
☒	☐	A full description of the statistical parameters including central tendency (e.g. means) or other basic estimates (e.g. regression coefficient) AND variation (e.g. standard deviation) or associated estimates of uncertainty (e.g. confidence intervals)
☐	☒	For null hypothesis testing, the test statistic (e.g. *F*, *t*, *r*) with confidence intervals, effect sizes, degrees of freedom and *P* value noted<br>*Give P values as exact values whenever suitable.*
☒	☐	For Bayesian analysis, information on the choice of priors and Markov chain Monte Carlo settings
☒	☐	For hierarchical and complex designs, identification of the appropriate level for tests and full reporting of outcomes
☒	☐	Estimates of effect sizes (e.g. Cohen's *d*, Pearson's *r*), indicating how they were calculated

Our web collection on statistics for biologists contains articles on many of the points above.

Software and code

Policy information about availability of computer code

Data collection	No software was used
Data analysis	GraphPad Prism v9.1 (Statistical Analysis)

For manuscripts utilizing custom algorithms or software that are central to the research but not yet described in published literature, software must be made available to editors and reviewers. We strongly encourage code deposition in a community repository (e.g. GitHub). See the Nature Portfolio guidelines for submitting code & software for further information.

Data

Policy information about availability of data

All manuscripts must include a data availability statement. This statement should provide the following information, where applicable:

- Accession codes, unique identifiers, or web links for publicly available datasets
- A description of any restrictions on data availability
- For clinical datasets or third party data, please ensure that the statement adheres to our policy

16S rRNA gene sequencing and bacterial strain genome sequencing data have been deposited in the ENA (European Nucleotide Archive, https://www.ebi.ac.uk/ena) under the accession number: PRJEB74255 and PRJEB42167, respectively. Furthermore, we have deposited our LC-MS/MS data in the MassIVE repository (https://massive.ucsd.edu) under the accession number: MassIVE ID MSV000095337. Source data are provided with this paper.

Research involving human participants, their data, or biological material

Policy information about studies with human participants or human data. See also policy information about sex, gender (identity/presentation), and sexual orientation and race, ethnicity and racism.

Reporting on sex and gender	*Use the terms sex (biological attribute) and gender (shaped by social and cultural circumstances) carefully in order to avoid confusing both terms. Indicate if findings apply to only one sex or gender; describe whether sex and gender were considered in study design; whether sex and/or gender was determined based on self-reporting or assigned and methods used. Provide in the source data disaggregated sex and gender data, where this information has been collected, and if consent has been obtained for sharing of individual-level data; provide overall numbers in this Reporting Summary. Please state if this information has not been collected. Report sex- and gender-based analyses where performed, justify reasons for lack of sex- and gender-based analysis.*
Reporting on race, ethnicity, or other socially relevant groupings	*Please specify the socially constructed or socially relevant categorization variable(s) used in your manuscript and explain why they were used. Please note that such variables should not be used as proxies for other socially constructed/relevant variables (for example, race or ethnicity should not be used as a proxy for socioeconomic status). Provide clear definitions of the relevant terms used, how they were provided (by the participants/respondents, the researchers, or third parties), and the method(s) used to classify people into the different categories (e.g. self-report, census or administrative data, social media data, etc.) Please provide details about how you controlled for confounding variables in your analyses.*
Population characteristics	*Describe the covariate-relevant population characteristics of the human research participants (e.g. age, genotypic information, past and current diagnosis and treatment categories). If you filled out the behavioural & social sciences study design questions and have nothing to add here, write "See above."*
Recruitment	*Describe how participants were recruited. Outline any potential self-selection bias or other biases that may be present and how these are likely to impact results.*
Ethics oversight	*Identify the organization(s) that approved the study protocol.*

Note that full information on the approval of the study protocol must also be provided in the manuscript.

Field-specific reporting

Please select the one below that is the best fit for your research. If you are not sure, read the appropriate sections before making your selection.

☒ Life sciences ☐ Behavioural & social sciences ☐ Ecological, evolutionary & environmental sciences

For a reference copy of the document with all sections, see nature.com/documents/nr-reporting-summary-flat.pdf

Life sciences study design

All studies must disclose on these points even when the disclosure is negative.

Sample size	Sample size for mouse studies was chosen according to institutional directives and in accordance with the 3Rs rules (Replacement, Reduction and Refinement) guiding principles underpinning the humane use of animals in research, but no statistical analyses were performed to predetermine the sample sizes.
Data exclusions	No data were excluded from the study.
Replication	All attempts at replication were successful, with multiple mice in each group (see sample size above). Some animal experiments were not replicated, but all experimental groups consisted of at least 3 animals.
Randomization	Mice were randomly allocated to different treatments.
Blinding	The investigators were not blinded to group allocation as the investigators needed to identify the cages of mice for subsequent colonization resistance tests.

Reporting for specific materials, systems and methods

We require information from authors about some types of materials, experimental systems and methods used in many studies. Here, indicate whether each material, system or method listed is relevant to your study. If you are not sure if a list item applies to your research, read the appropriate section before selecting a response.

Materials & experimental systems

n/a	Involved in the study
☒	☐ Antibodies
☒	☐ Eukaryotic cell lines
☒	☐ Palaeontology and archaeology
☐	☒ Animals and other organisms
☒	☐ Clinical data
☒	☐ Dual use research of concern
☒	☐ Plants

Methods

n/a	Involved in the study
☒	☐ ChIP-seq
☒	☐ Flow cytometry
☒	☐ MRI-based neuroimaging

Animals and other research organisms

Policy information about studies involving animals; ARRIVE guidelines recommended for reporting animal research, and Sex and Gender in Research

Laboratory animals	C57BL/6N SPF-H mice were bred and maintained at the in-house animal facility of the Helmholtz Centre for Infection Research (HZI) under specific pathogen-free (SPF) conditions. Germ-free (GF) C57BL/6NTac mice were bred in isolators in the GF facility at the HZI. Mice were kept under 12-hour light cycle (lights on from 7.00 am – 7.00 pm) and housed in groups of 3-6 mice per cage. Sterilized food and water was available to all animals ad libitum. Experimental animals were all age- and sex-matched, between the ages of 8 and 16 weeks. The number and sex of all experimental animals are indicated in Supplementary Data 4. GF mice were kept in airtight and individually ventilated ISOcages to prevent contamination. All mice were euthanized by asphyxiation with CO_2 and cervical dislocation.
Wild animals	The study did not involve wild animals
Reporting on sex	For all animal experiments, groups were age- and gender-matched.
Field-collected samples	This study did not involve samples collected from the field.
Ethics oversight	All animal experiments were performed in agreement with the guidelines of the Helmholtz-Zentrum für Infektionsforschung, Braunschweig, Germany, the national animal protection law (Tierschutzgesetz (TierSchG)), the animal experiment regulations (Tierschutz- Versuchstierverordnung (TierSchVersV)), and the recommendations of the Federation of European Laboratory Animal Science Association (FELASA). This study was approved by the Lower Saxony State Office for Nature, Environment and Consumer Protection (LAVES), Oldenburg, Lower Saxony, Germany; permit No. 33.19-42502-04-21/3818 and 33.33-42502-04-21/3795.

Note that full information on the approval of the study protocol must also be provided in the manuscript.
